# Given that the detailed original criteria for deliberate practice have not changed, could the understanding of this complex concept have improved over time? A response to Macnamara and Hambrick (2020)

**DOI:** 10.1007/s00426-020-01368-3

**Published:** 2020-06-24

**Authors:** K. Anders Ericsson

**Affiliations:** Department of Psychology, Florida State University, Tallahassee, FL 32306-1270 USA

## Abstract

In their commentary, Macnamara and Hambrick (Psychol Res, 2017) accused my colleagues and me of systematically changing the definition of the concept of deliberate practice. Deliberate practice was the result of a search for characteristics of effective practice in the laboratory that was shown to improve expert professional performance in domains, such as music. In this reply, I will first describe five different criteria that defined the original concept of deliberate practice and each of them is presented with directly supporting quotes from Ericsson, Krampe, and Tesch-Römer (Psychol Rev 100:396–406, 10.1037/0033-295X.87.3.215, 1993) paper. Unfortunately, Macnamara, Hambrick, and Oswald (Psychol Sci 25:1608–1618, 10.1177/0956797614535810, 2014) misinterpreted our concept of deliberate practice, and defined it much more broadly: “as engagement in structured activities created specifically to improve performance in a domain” (p. 914). This definition led them to include activities, such as attending lectures, studying alone by students, and group activities led by a coach, where each activity does not meet one or more of our criteria for deliberate practice. In this commentary, I will argue that Macnamara and Hambrick (2020) became aware of some of the original criteria for deliberate practice, such as the role of individualized training by a teacher, and these discoveries misled them to assume that we had changed our definition. The intended meaning of sentences that Macnamara and Hambrick (2020) had carefully selected is shown to have an appropriate interpretation in Standard English that is consistent with our original definition of deliberate practice. In conclusion, I will give a proposal for how the different perspectives can be reconciled.

## Introduction

In their commentary, Macnamara and Hambrick (2020) generously accepted the empirical findings and the central role of purposeful practice in attained SCRABBLE performance reported by Moxley, Ericsson, and Tuffiash (2017) but they criticized perceived contradictions in our definition of deliberate practice. They stated: “First and foremost, the authors have defined deliberate practice in flatly contradictory ways.” (p. 2).

To address their criticism, I will first document and describe our original definition of deliberate practice published in 1993 (Ericsson, Krampe, & Tesch-Römer, 1993) and how this type of practice differs from traditional conceptions of practice and accumulated experience by the simultaneous presence of five distinct characteristics. I will then compare this description to the definition that was used to include studies in their influential meta-analysis (Macnamara, 2014; Macnamara, Hambrick, & Oswald, 2014). I will show that what Macnamara and Hambrick (2020) claim to be inconsistencies and contradictions in our original definition of deliberate practice can be accounted for by an original misunderstanding of our definition and subsequent discoveries of differences between their incorrect definition and our original definition of deliberate practice. Once our field recognizes that these two definitions are distinctly different from each other and deserve unique names, I will propose how it should be possible to pursue research on expert performance in a cumulative manner.

## The original definition of deliberate practice published in 1993

The goal guiding Ericsson et al.’s (1993) original research was to search for effective training activities for attaining expert levels of performance in professional domains outside the laboratory. Ericsson et al. (1993) begin their paper by reviewing studies of effective learning in the laboratory conducted in the nineteenth and twentieth centuries and found conditions, where large improvement of performance with practice was consistently found: “The most cited condition concerns the subjects' motivation to attend to the task and exert effort to improve their performance. In addition, the design of the task should take into account the preexisting knowledge of the learners so that the task can be correctly understood after a brief period of instruction. The subjects should receive immediate informative feedback and knowledge of results of their performance. The subjects should repeatedly perform the same or similar tasks” (Ericsson et al., 1993, p. 367).

The key features of these conditions for improving performance are:

1. The task must be well defined with a clear goal and be fully understood by the participant.
2. The participants need to be able to perform the task by themselves
3. The participants need to gain immediate informative and actionable feedback on each performance of the practice task that allow them to make appropriate adjustments to improve.
4. The participant needs to be able to “repeatedly perform the same or similar tasks”
  Ericsson et al. (1993) go on to describe a fifth criterion:
5. The practice task must be designed and performed in accordance with individualized instruction and guidance of a teacher.

“To assure effective learning, subjects ideally should be given explicit instructions about the best method and be supervised by a teacher to allow individualized diagnosis of errors, informative feedback, and remedial part training. The instructor has to organize the sequence of appropriate training tasks and monitor improvement to decide when transitions to more complex and challenging tasks are appropriate” (p. 367). These criteria were explicitly described in my immediate unpublished response (Ericsson 2014a) to Macnamara et al.’s (2014) meta-analysis and this response was explicitly cited by Hambrick, Macnamara, Campitelli, Ullén, and Mosing (2016). The criteria were also described in my published criticism (Ericsson, 2016), as well as in earlier studies to be discussed later in this reply.

After a search for activities meeting the 5 criteria among domains of expertise with centuries of experience of successfully training individuals to attain reproducibly superior (expert) performance, Ericsson et al. (1993) focused on training at an international music academy, where teachers work individually with music students and state: “Throughout development toward expert performance, the teachers and coaches instruct the individuals to engage in practice activities that maximize improvement. Given the cost of individualized instruction, the teacher designs practice activities that the individual can engage in between meetings with the teacher. We call these practice activities *deliberate practice”* (p. 368, italics in the original).

When a practice activity meets all of the five external conditions (criteria) we referred to it as deliberate practice. Ericsson et al. (1993) also discussed efforts to find practice activities in other domains that met only some, but not all of these conditions (criteria) in domains of expertise, such as chess, typing, and sports. Ericsson (2009) is rather explicit about the fact that only some criteria for deliberate practice are met for some activities: “It has been more difficult to isolate practice activities that meet *all* the criteria for deliberate practice.” (p. 419, italics added). Similarly, in the same edited volume, Ericsson et al. (2009) referred to “the amount and quality of solitary activities *meeting the criteria of deliberate practice* and performance in different domains of expertise” (p. 9, italics added), they intended to say “some of the criteria” and definitely not “all of the criteria”. I agree that this sentence would have removed any ambiguity if it had included “some of”, but Macnamara and Hambrick’s claim that it implicitly says “all of” is not accurate given that no reference to these criteria had been made earlier in this chapter, the use of the definite article, “the” has no known referent and, thus, can refer to any combinations of criteria for deliberate practice.

The central claim of Macnamara and Hambrick’s (2020, p. 4) commentary is that Moxley et al. (2017) “replace[d} the term ‘deliberate practice’ with ‘purposeful practice’”, when they described the same solitary practice with SCRABBLE reported by Tuffiash, Roring, & Ericsson (2007). A more careful reading shows that Tuffiash et al. (2007) identified some practice activities that met several of the criteria, but not all, for deliberate practice, which they described as “the quantity of time spent on SCRABBLE-related activities that *best met the theoretical description of deliberate practice*” (p. 131, emphasis added). Consequently, Moxley et al. (2017) did not change any definitions, but used the name, purposeful practice, for those practice activities that met the first four criteria, but not the fifth criterion involving individualized guidance by a qualified teacher.

Before discussing some of the other quoted sentences alleged to be inconsistent with our original definition of deliberate practice, I will propose one possible alternative explanation for why Macnamara and Hambrick (2020) perceived inconsistencies in our definition of deliberate practice.

## The definition of practice adopted by Macnamara and Hambrick in 2014

Macnamara et al.’s (2014) meta-analysis was the first joint publication by both Macnamara and Hambrick and the first joint paper with a proposed definition of “deliberate practice”. In this paper, Macnamara et al. (2014, p.1608) described their definition of this concept: “deliberate practice, which Ericsson et al. defined as engagement in structured activities created specifically to improve performance in a domain.” In their meta-analysis, “the first formal meta-analysis of the relationship between deliberate practice and human performance” (p. 1609), they report identifying 9331 potentially relevant studies and selecting 88 studies for inclusion. The only inclusion criterion directly referencing “deliberate practice” and it required that: “a measure of accumulated amount (e.g., number of hours) of one or more activities interpretable as deliberate practice (henceforth, *deliberate practice*) was collected, with reference in the study report to at least one publication on deliberate practice by Ericsson et al.” (Macnamara et al., 2014, p. 1610, bold added).

Their definition specifies that the practice activity needs to be structured, but that does not seem to rule out practice activities with the exception of play. The more informative part of that definition states that the activity has to be designed to improve performance, but does not specify by whom it was designed: the performers themselves or their teachers or coaches? In contrast, our definition of deliberate practice avoids these problems of ambiguity and is a conjunction of five criteria expressed in terms of standard concepts in psychology. These concepts are reasonably invariant across time, such as “explicit goals for improving performance”, “immediate feedback”, “opportunity to repeat”, and “guidance by teachers”, although further theoretical and empirical refinement in measurement of each of these concepts is both likely and desirable.

To distinguish their definition from ours in the following text, I will now refer to their definition of relevant practice activities, as *structured practice*. An examination of the practice activities that they included in their meta-analysis shows them to be consistent with their definition of structured practice because the activities are both structured as well as designed by somebody to improve performance. For example, they included a study where the structured practice involved attending lectures (Masui, Broeckmans, Doumen, Groenen, & Molenberghsm, 2012). This activity is clearly structured and led by a teacher. The lecture is designed by a teacher to improve the students’ performance. This activity, however, does not meet the criteria requiring individualized assessment of each students’ current performance and the individualized design of appropriate practice activities for each student, nor would the lecture typically include practice activities that can be repeatedly performed with clear goals and immediate actionable feedback. Other structured practice activities included in their meta-analysis involve watching games relevant to their sport on tv (Baker, Côté, & Abernathy, 2003), flying airplanes (McKinney, & Davis, 2003), studying at home by middle-school students (Rosário, Núñez, Valle, González-Pienda, & Lourenço, 2013) and practice of a soccer team under the direction of a coach (Hendry, 2012). Although soccer practices are led by a coach, this type of practice is not individualized to address each individual players’ weaknesses and needs.

Somebody might argue that the requirement of only including studies citing Ericsson and colleagues restricted the inclusion to data of deliberate practice. However, 45 effect sizes (88% of the effect sizes in the domain of Education) came from studies that never used the term “deliberate practice” anywhere in the text of their articles. Their only mention of deliberate practice was in the reference section as part of the title of Plant, Ericsson, Hill and Asberg’s (2005) paper. The main conclusion of Plant et al.’s (2005) paper was that the hours of study time by students did NOT measure effective practice and that it differed from deliberate practice.

Another argument that Macnamara & Hambrick (2020) might make is that in the discussion of differences of deliberate practice compared to play and work, Ericsson et al. (1993) even wrote: “In comparison to play, deliberate practice is a highly structured activity, the explicit goal of which is to improve performance” (p. 368). A few sentences later the same text states that deliberate practice “is not enjoyable” (p. 368). The first sentence was, however, not intended by us to be a definition of deliberate practice, not any more than the second sentence. Consequently, all activities that are not enjoyable, such as falling during a gymnastics routine and putting ice on sore limbs, do not meet the criteria for deliberate practice.

In a recent effort to explicate consequences of conducting a meta-analysis of the relation to achievement based on different definitions of accumulated structured versus deliberate and purposeful practice, Ericsson & Harwell (2019) examined all the studies included in Macnamara et al.’s (2014) meta-analysis, but they restricted entry to only those studies that met criteria for purposeful and deliberate practice. In addition, their meta-analysis excluded two cases where Macnamara et al. (2014) included the same data set more than once, when the same data were included in the analyses in two different publications. Ericsson & Harwell’s (2019) meta-analysis found that the relation between attained performance and purposeful and deliberate practice was substantially stronger than those reported by Macnamara et al. (2014), and these variables accounted for over 50% of the variance in performance after attenuation. Macnamara and Hambrick were both co-authors to a recent analysis which showed that over 50% of the variance in peak chess rating could be accounted for by variables related to practice (Burgoyne, Nye, Macnamara, Charness, & Hambrick, 2019).

## The origin of the definition of structured practice in Macnamara et al. (2014)

The first public report that described the meta-analysis, which was eventually published in *Psychological Science,* appeared as Chapter 6 in Macnamara’s (2014) doctoral dissertation. The scope of her dissertation was impressive and included a couple of longitudinal studies, a pair of experimental studies, and one meta-analysis that each examined determinants of performance with subtitle “Cognitive Abilities, Experiential Factors and Predictability of the Task Environment” (p. i).

The first two studies of her dissertation examined the longitudinal changes of performance, where accumulated experience was measured by traditional indicators, such as the number of years working as a translator or the number of years of study toward becoming a translator by students. In Chapter 5 of her dissertation, she mentions deliberate practice, which provides some insights into her understanding of the concept. In one of her experimental studies, participants were playing a video game, and prior to each training block, the experimenter gave the same information about a character in the game to all participants, The participants were instructed to use that information while simply playing the game by themselves for a fixed time period to improve their performance. It is notable that she referred to each block of practice as a deliberate practice block. In the abstract to her dissertation, she described her final study, which examined whether her findings on predictability of task environment “translated into real-world domains, a meta-analysis was conducted examining the relationship between *practice* and performance variance.” (Macnamara, 2014, p. v, italics added).

In Chapter 6, she reports on her meta-analysis and describes her search for studies of practice and performance. She defined the targeted type of practice (structured practice) “as engagement in structured activities created specifically to improve performance in a domain” (Macnamara, 2014, p. 110). Her criteria for inclusion of a study were that it studied “practice activities *interpretable* as deliberate practice” (Macnamara, 2014, p. 114, italics added) and the need for citations to the research by myself and collaborators. Her initial search found 9331 papers and she identified 88 papers that met the above criterion and a few other criteria unrelated to the type of practice. It appears that she was the only person, who selected the 88 papers because no additional information is given, but considerable detail is given on how the mediator variables were coded for the 88 papers. The meta-analysis reported in her dissertation was reproduced without any changes in Macnamara et al. (2014), which implies that her co-authors agreed with her selection of studies.

In sum, an alternative account of Macnamara and Hambrick (2020) accusation that my colleagues and I changed our definition of deliberate practice would suggest that Macnamara (2014) misrepresented our original definition of deliberate practice. Macnamara et al. (2014, p. 1608) clearly stated that they wanted to rely on our original definition of “deliberate practice, which Ericsson et al. defined as engagement in structured activities created specifically to improve performance in a domain”, where “Ericsson et al.” refers to Ericsson et al. (1993) in the previous sentence. Based on the assumption made by Macnamara and Hambrick (2020) that their definition of structured practice was the correct interpretation of our original definition of deliberate practice, it is understandable they might have perceived our introduction of the new theoretical terms, naïve and purposeful practice, as involving changes to our original definition, as they had incorrectly understood it.

## “Theoretical Term Swapping” versus discovery of overlooked aspects of the original definition of deliberate practice

The original definition of deliberate practice has five criteria but Macnamara and Hambrick (2020) focus almost exclusively on a single criterion, namely the necessity that a teacher or a coach guides the practice activities, They cite sentences stating, for example that “activities can be designed by external agents, such as teachers or trainers, or by the performers themselves.” (Keith & Ericsson, 2007, p. 136) to argue that we argue that deliberate practice “does *not* require a teacher”. Being a non-native speaker of English, I turned to academic papers on the meaning of “or”. Dickerson (1960) insists that the default interpretation of “or” is as “and/or” rather than the exclusive “or”. By giving the sentence above an interpretation in terms of an exclusive “or”, Macnamara and Hambrick (2020) argue that any practice activity where individuals attempt to acquire skilled performance by deciding what to do during practice (designing their own practice activities) by themselves is a legitimate example of structured practice. It is understandable that somebody who believes that structured practice is equivalent to deliberate practice might be confused when Moxley et al. (2017) argue that self-study in SCRABBLE is not deliberate practice but rather an example of purposeful practice. As shown earlier, the original definition of deliberate practice remains unchanged and purposeful practice refer to a practice activity that meets the first four criteria of deliberate practice, but lacks individualized training guided by a teacher or coach.

## Challenges related to assessing individualized training guided by a teacher or a coach

In our original paper, Ericsson et al. (1993) studied highly motivated advanced students in a famous music academy. These participants viewed “solitary practice” as the most relevant practice activity for improving performance. When music students work individually with their respective master teacher, practice alone provide the external preconditions for highly effective learning. In a recent paper (Ericsson, 2020), I expanded earlier observations that the external conditions do not by themselves cause improvement. I discussed findings that some young music students did not improve performance during solitary practice even though they were supervised by a teacher. Analyses of videotape of the practice of these piano students showed that they were unable or not sufficiently motivated to engage with full concentration on attaining the assigned goals, and they kept hitting the same sequence of keys over and over. Another limitation is that motivated students studying with an experienced teacher of reading Tarot cards, who engage in their assigned practice are very unlikely to increase the accuracy of their predictions about their clients’ future. In our theoretical framework, the teacher is an agent communicating accumulated knowledge about how learning can best proceed in a given domain. “In all major domains, there has been a steady accumulation of knowledge about the best methods to attain a high level of performance and the associated practice activities leading to this performance. Full-time teachers and coaches are available for hire and supervise the personalized training of individuals at different levels of performance starting with beginners.” (Ericsson et al., 1993, p. 368). To implement the fifth criterion for deliberate practice, the type of support provided by teachers will differ as function of age and attained skill level of the trainee.

In domains with individualized instruction such as a child's first introduction to the domain of music performance, this guidance by teachers might be very direct, step by step. However, with acquired skill music students develop their own mental representations, so they can image the sound of music that they cannot yet produce. This allows them to generate iteratively an actual music performance that matches their original image of the desired music experience (Ericsson & Harwell, 2019). When the master teachers interact with highly skilled music students, they will provide higher-level goals on how a segment of a given music piece can be played with more emphasis, tone, speed, and volume. During the solitary practice, the students will engage in problem solving and refine the particular practice activities that eventually permits them to play the music piece in the recommended manner. This type of complex interactive practice activity seemed best described as designed by the teachers or the performers themselves, where “or” is interpreted as “and/or”. In music and many other domains, the goal of training of beginners and intermediates is develop their mental representations so that they become skilled performers that are capable of generating many of the improvements by practicing alone.

In sum, the individualized training by teachers help the students to acquire correct fundamentals as beginners and encourage them to refine their mental representations; the accumulated number of hours of deliberate practice, therefore, is predicted to be related (correlated) with attained performance. However, future research is likely to allow descriptions of the longitudinal development in terms of each of the individuals’ practice sessions and the attained performance improvements. With these refined descriptions, it should be possible in the future to identify more of the contributions of practice to final level of achievement (Ericsson, 2016, 2020).

The relations between accumulated amount of deliberate practice on expert music performance (Ericsson et al., 1993) and the large effects of extended practice on many types of performance (Ericsson, Chase, & Faloon, 1980; Howe, Davidson, & Sloboda, 1998) raised the issue of searching for but not finding research studies directed towards measuring the role of genetic factors in the acquisition of high levels of performance (Plomin, 1998)—even more recent reviews of newer studies have not found evidence for substantial genetic contributions (Ericsson, 2014b, 2016, 2020). Macnamara et al. (2014) took a different approach and estimated how much of individual differences in attained performance could be accounted for estimates of accumulated hours of structured practice and argued that most of the remaining unexplained variance must reflect other factors than practice. These sums of hours of practice do not estimate the maximal accounts of performance by practice and the accounted variance is likely to greatly increase in the future with more refined measures of different types of practice. Our framework does not categorically reject any influence of genetic differences, but it requires that the predictive roles of particular genes and their interactions have to be demonstrated in the same way that rough measures of individual differences in practice have been able to do. In fact, our original paper (Ericsson et al., 1993) reviewed compelling evidence for the role of inherited genetic factors in determining height and their influence on success in sports, such as basketball. Although we did not find evidence for an important role of innate unmodifiable cognitive capacities developing independently without engagement in any activities (cf. height, our paper clearly stated: “It is quite plausible, however, that heritable individual differences might influence processes related to motivation and the original enjoyment of the activities in the domain and, even more important, affect the inevitable differences in the capacity to engage in hard work (deliberate practice)” (Ericsson et al., 1993, p. 399).

## Concluding remarks

Macnamara and Hambrick (2020, p. 5) summarize their view in their final paragraph: “The major finding from our own and others’ research on this topic is that deliberate practice, while certainly important, leaves a large amount of the inter-individual variability in expertise unexplained and *potentially explainable* by other factors” (p. 5. italics added). When I read this sentence, I am reminded of a similar conclusion in Ericsson et al.’s (1993, p. 400) paper in the absolutely last paragraph of the text of our paper with 41 pages: “We believe that a more careful analysis of the lives of future elite performers will tell us how motivation is promoted and sustained. It is also entirely plausible that such a detailed analysis will reveal environmental conditions as well as *heritable individual differences* that predispose individuals to engage in deliberate practice during extended periods and facilitate motivating them” (italics added). I look forward to new and further refined definitions and associated measures of amount and quality of different kinds of practice, that will take us beyond distinctions between naïve, purposeful, structured and deliberate practice and hopefully uncovering practice activities that will more effectively improve particular types of performance. This would allow the steady accumulation of empirical evidence to identify the best predictors of attained performance and performance improvements as I proposed in the final paragraph in Ericsson (2013, p. 535): “In the future we should be able to develop statistical models integrating genetic and training factors along with their interactions to account for the attained performance level of elite athletes”.
